# Role of Contralesional Corticoreticulospinal Tract Compensation in Walking Function After Stroke

**DOI:** 10.1002/brb3.71500

**Published:** 2026-05-27

**Authors:** Jolene Foster, Oluwole O. Awosika, Pierce Boyne

**Affiliations:** ^1^ Department of Rehabilitation, Exercise, and Nutrition Sciences, College of Allied Health Sciences University of Cincinnati Cincinnati Ohio USA; ^2^ Department of Neurology and Rehabilitation Medicine, College of Medicine University of Cincinnati Cincinnati Ohio USA

**Keywords:** brain, gait, locomotion, stroke

## Abstract

**Introduction:**

Evidence suggests the contralesional corticoreticulospinal tract (cCRST) upregulates after stroke and that this upregulation correlates with worse motor function, suggesting it may be harmful for walking function. However, this relationship may be confounded by the extent of ipsilesional corticospinal tract (CST) and CRST damage, which could cause both greater cCRST upregulation and worse walking function. No previous studies have tested whether this confounding relationship exists, nor whether the amount of damage to the ipsilesional motor tracts is related to the amount of cCRST upregulation. We hypothesized that lower ipsilesional motor tract strength would (1) be associated with greater cCRST strength and (2) explain the observed association between cCRST strength and walking function.

**Methods:**

Ten individuals with chronic stroke and 10 age‐ and sex‐matched controls completed diffusion MRI, from which quantitative anisotropy was derived to evaluate the strength of the ipsilesional and contralesional CRST and CST. Walking capacity was assessed using 6‐min walk distance (6MWD). Linear regressions were applied to examine relationships among ipsilesional corticomotor tract strength (iCRST and iCST combined), cCRST strength, and 6MWD.

**Results:**

Compared with controls, participants with stroke had lower ipsilesional and higher contralesional strength for both motor tracts. Lower ipsilesional tract strength was associated with greater cCRST strength *z*‐score (−0.12 SDs [−0.23, −0.02]). The unadjusted association between greater cCRST strength *z*‐score and lower walking capacity (−72 m [−136, −9]) was no longer present after adjusting for ipsilesional tract strength (−3 m [−28, 23]).

**Conclusions:**

Greater damage to ipsilesional motor tracts (lower strength) was associated with increased cCRST strength. The extent of ipsilesional tract injury fully explained the negative association between cCRST strength and worse walking capacity. These findings suggest that cCRST upregulation may be an adaptive compensation; however, larger studies are needed to better understand the impact of cCRST upregulation on walking function.

**Trial Registration:**

ClinicalTrials.gov identifier: NCT02858349

## Graphical abstract

This study assessed the association between contralesional corticoreticulospinal tract (cCRST) upregulation and walking capacity, while controlling for ipsilesional motor tract damage in participants with chronic stroke. Ipsilesional tract damage fully explained the negative association between cCRST strength and worse walking capacity, suggesting that cCRST upregulation may be an adaptive compensation.

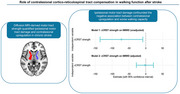

**Abbreviations**
6MWD: 6‐min walk distance
cCRST: contralesional corticoreticulospinal tract

## 1 Introduction

Despite targeted locomotor interventions, an estimated 80% of patients still experience limitations in walking function after stroke (Beyaert et al. 2015), including reduced walking capacity (i.e., speed and endurance), which affects their ability to participate in daily activities (Mayo et al. 2002; Rudberg et al. 2021). A better understanding of how brain pathways reorganize in relation to walking capacity after stroke may lead to improved patient outcomes by guiding interventions such as noninvasive brain stimulation (NIBS), which enhances neural mechanisms involved in recovery. While NIBS has shown promise for improving walking outcomes after stroke, more evidence is needed to support decision‐making on which hemisphere and neural tracts to target, and whether to facilitate or inhibit those targets to support improved walking outcomes (Veldema and Gharabaghi 2022; Li et al. 2023).

The corticospinal tract (CST) is traditionally considered the primary descending motor tract driving functional movement in humans; however, patients with complete damage to the CST have demonstrated the ability to recover independent walking (Ahn et al. 2006; Jang et al. 2013; Jo et al. 2012; van Hinsberg et al. 2025; Yeo et al. 2020). These findings suggest other non‐CST neural tracts can contribute to walking function after stroke. Accumulating evidence suggests that the corticoreticulospinal tract (CRST) may play a central role in poststroke walking function. For example, white matter integrity of the ipsilesional CRST (iCRST) appears to be strongly correlated with walking independence and capacity (Jang et al. 2022; Srivastava et al. 2022). Unlike the CST, the CRST also projects more bilaterally to the brainstem and spinal cord and is mainly ipsilateral, providing an anatomical basis for the contralesional hemisphere to compensate for ipsilesional tract damage (Boyne, DiFrancesco, et al. 2022).

Evidence also suggests that the contralesional CRST (cCRST) may be upregulated after stroke (Jang et al. 2013; Srivastava et al. 2022; Karbasforoushan et al. 2019), but it remains unclear whether this is helpful or harmful. While greater cCRST strength (i.e., upregulation) has been associated with worse motor function (Karbasforoushan et al. 2019), this association could be confounded by stroke severity rather than reflecting a maladaptive role for cCRST upregulation. In other words, this association could be explained by greater ipsilesional damage causing both greater compensatory cCRST upregulation and worse motor function. This aligns with the bimodal balance recovery model proposed by Di Pino et al. (2014), which suggests that the brain's response to stroke depends on the extent of residual structural integrity in ipsilesional motor pathways. According to this model, individuals with higher structural reserve (i.e., less damage) are more likely to recover through restoration of ipsilesional motor pathways, while those with lower structural reserve (i.e., more damage) may rely more on compensatory mechanisms involving the contralesional hemisphere (Di Pino et al. 2014). In this context, cCRST upregulation may represent an adaptive, compensatory response in individuals with more severe ipsilesional damage, rather than a maladaptive response.

Some supporting evidence for this can be found in one study that limited the sample to only those with complete ipsilesional motor tract damage, partly controlling for this confounding. In this study, greater cCRST upregulation was indeed associated with a higher motor function and a greater likelihood of achieving independent walking (Jang et al. 2013). However, no previous studies have tested whether this confounding relationship exists or whether the extent of motor tract damage is associated with the amount of cCRST upregulation. Understanding this relationship is essential for determining whether cCRST upregulation is a compensatory response and could provide insights to inform future interventions.

This study aimed to assess whether cCRST motor tract strength is a compensatory response by testing (1) whether cCRST motor tract strength upregulation magnitude relates to ipsilesional motor tract strength and (2) whether the relationship between cCRST motor tract strength and walking function is confounded by ipsilesional motor tract strength. We hypothesized that lower ipsilesional motor tract strength would (1) be associated with greater cCRST motor tract strength and (2) account for the observed relationship between cCRST strength and walking function.

## 2 Materials and Methods

### 2.1 Study Design

The study providing data for this analysis was approved by institutional review boards, preregistered on ClinicalTrials.gov (NCT02858349), and performed in a cardiovascular stress laboratory, magnetic resonance imaging (MRI) research center, and rehabilitation research laboratory from July 2016 to December 2017. Some of the methods and baseline data from this study have been previously described in manuscripts addressing different aims from the current report (Boyne et al. 2020, 2021; Boyne, Doren, et al. 2022).

### 2.2 Participants

Ten participants with chronic stroke and 10 age‐ and sex‐matched controls were recruited from the community and provided written informed consent. Inclusion criteria for both groups were as follows: age 30–90 years old, MRI compatible, able to communicate with investigators and answer consent comprehension questions correctly, able to perform mental imagery (Malouin et al. 2008), no recent history of drug/alcohol abuse or significant mental illness, and not pregnant. Additional inclusion criteria specifically for participants with stroke were as follows: unilateral stroke >6 months prior to enrollment in the middle cerebral artery territory; walking speed <1.0 m/s on the 10 m walk test (Bohannon and Williams Andrews 2011); able to walk 10 m with an assistive device as needed and no physical assistance by another person; no evidence of significant arrhythmia or myocardial ischemia on a treadmill electrocardiogram (ECG) stress test; no significant baseline ECG abnormalities that would make an exercise ECG uninterpretable (American College of Sports Medicine 2014); no recent cardiopulmonary hospitalization; no significant ataxia or neglect (NIHSS item score >1) (Brott et al. 1989); no severe lower extremity (LE) hypertonia (Ashworth >2) (Ashworth 1964); no major poststroke depression (PHQ‐9 ≥10) (Williams et al. 2005) in the absence of management by a health care provider (Duncan et al. 2007); not participating in physical therapy or another interventional research study; no recent paretic LE botulinum toxin injection; and no other progressive neurologic disorder or other major conditions that would limit capacity for improvement. Control participants were a demographic match for a participant with stroke (sex, age difference ≤5 years) without any current neurologic, orthopedic, or medical condition affecting walking function.

### 2.3 Walking Capacity Assessment

Walking capacity was measured by the 6‐min walk distance (6MWD). During the test, the participant is instructed to walk as far as possible in 6 min using a standardized course (ATS Committee on Proficiency Standards for Clinical Pulmonary Function Laboratories 2002). The distance walked provides a reliable and valid measure of walking capacity and is associated with community ambulation after stroke (Fulk et al. 2008, 2011). A blinded rater assessed the test.

### 2.4 MRI Data Acquisition

A 3.0T Philips Ingenia MRI system was used. T1‐weighted brain images were acquired at 1 mm isotropic resolution with the following parameters: TR, 8.1 ms; TE, 3.7 ms; flip angle, 8°; and SENSE factor 2. Diffusion‐weighted brain images were acquired at 2 mm isotropic resolution with the following parameters: TR, 7.1 s; TE, 92 ms; flip angle, 90°; 61 directions at *b* = 1000 s/mm^2^; seven *b* = 0 volumes; and SENSE factor 3.

### 2.5 MRI Data Preprocessing

For the T1 images, FSL software (Jenkinson et al. 2012) was used for bias field correction, tissue type segmentation, and nonlinear registration to the MNI152 template, using a lesion mask to improve registration for participants with stroke by temporarily filling the lesion with MNI template voxels (Siegel et al. 2017). Diffusion MRI preprocessing included eddy current correction, motion correction, and outlier volume replacement using FSL's “eddy” tool (Jenkinson et al. 2012; Andersson and Sotiropoulos 2016; Andersson et al. 2016). Alignment to the T1 image with boundary‐based registration was performed using the T1 white matter segmentation (Jenkinson et al. 2012; Zhang et al. 2001). Diffusion data were reconstructed with generalized Q sampling imaging using DSI Studio (https://dsi‐studio.labsolver.org/) with a diffusion length ratio of 1.7 to calculate quantitative anisotropy (QA) in each brain voxel (Yeh 2025; Yeh et al. 2010).

Diffusion anisotropy metrics such as fractional anisotropy (FA) and QA are commonly used as markers of tract strength to assess residual microstructural integrity and neural reorganization processes after stroke (Yeh et al. 2013; Pinter et al. 2020; Loubinoux et al. 2024). Lower anisotropy is associated with greater white matter damage and motor impairment, whereas higher anisotropy reflects better microstructural integrity, organization, and axonal remodeling (Pinter et al. 2020; Loubinoux et al. 2024; Stinear et al. 2007; Wen et al. 2016). While FA is the most widely used anisotropy metric, it has limitations. For example, FA is sensitive to extracellular water (i.e., edema) and multiple fiber directions within a voxel, both of which make FA values underestimate axonal projection strength (Yeh et al. 2013). In contrast, QA is less susceptible to partial volume effects and offers improved resolution of multiple fiber directions, making it a more specific indicator of axonal projection strength (Yeh et al. 2013). In this study, we use QA of ipsilesional motor tracts as an inverted measure of ipsilesional motor tract damage and QA of the contralesional motor tracts to quantify neuroplasticity (i.e., contralesional upregulation).

### 2.6 Voxel‐Based Normalization

To improve comparability of diffusion metrics across participants, QA data were normalized (nQA) by dividing voxel‐wise QA values by the mean QA within ventricular cerebrospinal fluid (CSF) from the same scan (Fortin et al. 2016). CSF was selected as the normalization reference because it is not affected by pathology, and variability in CSF signals primarily reflects acquisition‐related variability (Fortin et al. 2016). nQA values were projected onto a standard space map of the core white matter “skeleton” using a method analogous to FSL's Tract‐Based Spatial Statistics (TBSS) (Smith et al. 2006). This approach helps correct residual misalignments after standard space registration, which is important after stroke because of tissue changes that complicate registration (Jühling et al. 2024; Andersen et al. 2010). For each voxel in the white matter skeleton, the highest nearby nQA value for the participant was projected onto the skeleton to better align the center of each white matter bundle to the standard space template (Smith et al. 2006).

### 2.7 Tract Strength Measurement

For each participant, mean nQA values were calculated within each tract (iCRST, cCRST, iCST, cCST). Using normative tract templates (Boyne et al. 2025), the contribution of each voxel to the mean nQA value was weighted by the number of streamlines passing through each voxel for that tract. The normative tract templates are available at Open Science Framework (https://osf.io/q3gw7/files/osfstorage). The specific links to each normative tract template used are listed below:

- Left CRST: https://osf.io/q3gw7/files/xjtue
- Right CRST: https://osf.io/q3gw7/files/bwe84
- Left CST: https://osf.io/q3gw7/files/mntsa
- Right CST: https://osf.io/q3gw7/files/9umnt

This calculation specifically used voxels in the internal capsule region (*z* = −5 to 12 mm) of each tract, as this region is less prone to diffusion MRI distortions (Tang et al. 2018). In addition, the internal capsule demonstrates high anisotropy and contains a dense concentration of descending motor fibers, making it a reliable region for tract‐specific quantification (Hua et al. 2008). Tract anisotropy within the internal capsule has also been associated with motor function after stroke, including the upper limb Fugl‐Meyer motor score (Stinear et al. 2007), change in Functional Independence Measure motor subscale (Wen et al. 2016), and a composite measure of upper limb impairment and function (Park et al. 2013).

The nQA measurement for each tract was then adjusted for the global mean cerebral white matter nQA for that participant to more specifically quantify the strength of that tract independent of global white matter strength. To do this, linear regression models were developed to quantify the relationship between global nQA (independent variable) and tract‐specific nQA (dependent variable), averaged across the left and right tracts. Only control participant data were used to quantify this relationship so that the resulting equations could be used to assess how the stroke survivor tract values differ from the expected normative value for someone with the same global QA as the participant, but without damage to that pathway. These models had regression slopes (*β*) of 1.186 and 2.702 and *r* ^2^ values of 0.735 and 0.883 for the CRST and CST, respectively. The regression slopes from these models and the mean global nQA across the control participants (2.658) were then used to adjust the tract nQA values for each participant, using the following formulas for both the ipsilesional and the contralesional tracts (the ipsilesional tract for controls was the same as their matched counterpart with stroke for all formulas):

(1) $$\begin{array}{ccc} \mathrm{Adjusted}\;\mathrm{CRST}\;{\mathrm{nQA}}_{i} & & =\mathrm{unadjusted}\;\mathrm{CRST}\;{\mathrm{nQA}}_{i}-1.186\times \\ & & \left(\mathrm{global}\;{\mathrm{nQA}}_{i}-2.658\right), \end{array}$$

(2) $$\begin{array}{ccc} \mathrm{Adjusted}\;\mathrm{CST}\;{\mathrm{nQA}}_{i} & & =\mathrm{unadjusted}\;\mathrm{CST}\;{\mathrm{nQA}}_{i}-2.702\times \\ & & \left(\mathrm{global}\;{\mathrm{nQA}}_{i}-2.658\right). \end{array}$$

In addition to calculating separate strength measures for each tract, we also quantified the overall strength of the motor pathways of interest within each hemisphere by calculating a composite corticomotor measure as the weighted average of the CRST and CST. Specifically, the ipsilesional corticomotor strength (iCorticomotor strength) was defined as the weighted average of the iCST strength and iCRST strength, and the contralesional corticomotor strength (cCorticomotor strength) was the weighted average of the cCST strength and cCRST strength. The contribution of each tract to this average was weighted by the mean normative volume of that tract, in order to account for differences in tract size (Boyne et al. 2025). Finally, to improve interpretability, tract nQA values were then converted to *z*‐scores by subtracting the control mean and dividing by the control standard deviation, using the following formulas:

(3) $$\mathrm{iCRST}\;\mathrm{strength}\;\left(\mathrm{zscor}e_{i}\right)=\left(\mathrm{iCRST}\;\mathrm{nQ}A_{i}-3.407\right)/0.357,$$

(4) $$\mathrm{cCRST}\;\mathrm{strength}\;\left(\mathrm{zscor}e_{i}\right)=\left(\mathrm{cCRST}\;\mathrm{nQ}A_{i}-3.093\right)/0.272,$$

(5) $$\mathrm{iCST}\;\mathrm{strength}\left(\mathrm{zscor}e_{i}\right)=\left(\mathrm{iCST}\;\mathrm{nQ}A_{i}-5.106\right)/0.718,$$

(6) $$\mathrm{cCST}\;\mathrm{strength}\;\left(\mathrm{zscor}e_{i}\right)=\left(\mathrm{cCST}\;\mathrm{nQ}A_{i}-4.862\right)/0.534,$$

(7) $$\begin{array}{ccc} & & \mathrm{iCorticomotor}\;\mathrm{strength}\;\left(\mathrm{zscor}e_{i}\right)\\ & & =\left(\mathrm{iCorticomotor}\;\mathrm{nQ}A_{i}-4.023\right)/0.145, \end{array}$$

(8) $$\begin{array}{ccc} & & \mathrm{cCorticomotor}\;\mathrm{strength}\;\left(\mathrm{zscor}e_{i}\right)\\ & & =\left(\mathrm{cCorticomotor}\;\mathrm{nQ}A_{i}-3.734\right)/0.169. \end{array}$$

We interpret higher values of motor tract strength for participants with stroke versus controls (i.e., a positive *z*‐score) as upregulation of that tract.

### 2.8 Data Analysis

Participant characteristics and tract strength values were compared between participants with stroke and controls using independent *t*‐tests. To address the hypotheses, linear regressions were applied to assess relationships between iCorticomotor strength, cCRST strength, and 6MWD. To assess whether contralesional cCRST upregulation is related to ipsilesional motor tract strength, the following regression was used:

(9) cCRSTstrength∼iCorticomotorstrength.

To assess whether the negative association between cCRST projection strength and 6MWD is confounded by ipsilesional motor tract strength, the following regressions were used:

(10) 6MWD∼cCRSTstrength,

(11) 6MWD∼cCRSTstrength+iCorticomotorstrength.

Confounding by the ipsilesional motor tract strength was determined to be present if adding iCorticomotor strength to the model shrank the regression coefficient for cCRST strength by more than 10% (from model 10 to 11) (Kleinbaum et al. 2014). To assess model stability, influence diagnostics were evaluated using Cook's distance (Cook 1977). An observation was determined to have a strong influence if its Cook's distance was greater than the *F* distribution quantile with *p* and *n* – *p* degrees of freedom, where *p* is the number of model predictors and *n* is the sample size (Cook 1977; Bollen 1985), yielding a threshold of *F*(3, 17) = 0.821. If a highly influential observation was detected, a leave‐one‐out sensitivity analysis was performed to assess the stability of the regression coefficients after removing that observation. All analyses were conducted using R version 4.4.1 (2025.9.0.387) (Posit Team n.d.).

### 2.9 Sample Size Calculation

The target sample size of 10 participants with stroke and 10 controls was calculated for different aims from those reported here (Boyne et al. 2021). That sample size provides 80% estimated power to detect (1) a within‐group Cohen's *d* effect size as small as 1.00; (2) a between‐group effect size as small as 1.32; and (3) a correlation as low as 0.58. These calculations were based on a two‐sided significance level of 0.05 and were performed with the R package “pwr” (Boyne et al. 2021).

## 3 Results

The target sample size (10 participants with stroke and 10 controls) was reached, and there were no missing data. Compared with controls, participants with stroke had significantly lower comfortable gait speeds and 6MWDs, but similar age and body mass index (Table 1).

**TABLE 1:** Participant characteristics.

	Stroke	Control	Stroke vs. control
	(*N* = 10)	(*N* = 10)	*p*‐value
Age (years)	59.8 ± 6.78	58.9 ± 7.79	0.79
Gender, *N* (%)			
Female	4 (40.0%)	4 (40.0%)	
Male	6 (60.0%)	6 (60.0%)	
Body mass index (kg/m^2^)	30.2 ± 4.24	30.8 ± 4.25	0.76
Six‐minute walk distance (m)	156 ± 147	537 ± 77.1	<0.0001
Years since stroke	2.4 ± 1.7	NA	
Type of stroke, *N* (%)			
Hemorrhagic	1 (10.0%)	NA	
Ischemic	9 (90.0%)	NA	
Lesion side, *N* (%)			
Left	5 (50.0%)	NA	
Right	5 (50.0%)	NA	
Lesion volume (mL)	122 ± 105	NA	
PHQ‐9 score	3.1 ± 3.1	NA	
Assistive device, *N* (%)			
Hemi walker	1 (10.0%)	0 (0%)	
Wide‐based quad cane	4 (40.0%)	0 (0%)	
Narrow‐based quad cane	2 (20.0%)	0 (0%)	
None	3 (30.0%)	10 (100%)	
Orthotics, *N* (%)			
AFO	8 (80.0%)	0 (0%)	
None	2 (20.0%)	10 (100%)	
Comfortable gait speed (m/s)	0.41 ± 0.33	1.36 ± 0.16	<0.0001

*Note*: Values are mean ± SD or *N* (%). The *p*‐values are from independent *t*‐tests.

Abbreviations: NA, not applicable; PHQ‐9, Patient Health Questionnaire‐9 total score.

**FIGURE 1:**
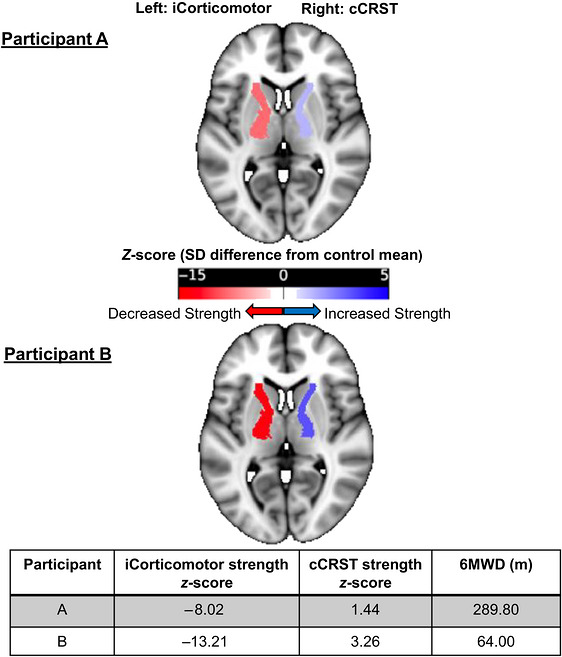
cCRST strength and 6MWD vary with iCorticomotor strength. Representative examples of tract strength quantification in two participants with differing iCorticomotor and cCRST strength profiles. Participant A demonstrates higher iCorticomotor strength with lower cCRST strength and greater walking capacity. Participant B demonstrates lower iCorticomotor strength with higher cCRST strength and reduced walking capacity. Tract strength was quantified using normalized quantitative anisotropy and expressed as *z*‐scores relative to controls. Negative values indicate lower tract strength, and positive values indicate higher tract strength relative to controls. iCorticomotor, weighted average of the ipsilesional corticospinal tract and ipsilesional corticoreticulospinal tract; cCRST, contralesional corticoreticulospinal tract; 6MWD, 6‐min walk distance.

Representative examples illustrating tract strength quantification are shown in Figure 1. Participant A demonstrated higher iCorticomotor strength, lower cCRST strength, and greater walking capacity, whereas Participant B demonstrated lower iCorticomotor strength, higher cCRST strength, and a reduced walking capacity.

**FIGURE 2:**
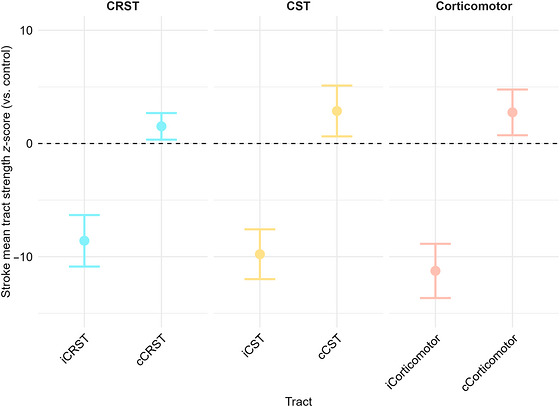
Stroke motor tract strength *z*‐scores relative to controls. Stroke *z*‐scores were calculated based on the mean and standard deviation in the control group and represent the number of standard deviations above or below the control mean value for that tract. CRST, corticoreticulospinal tract; iCRST, ipsilesional corticoreticulospinal tract; cCRST, contralesional corticoreticulospinal tract; CST, corticospinal tract; iCST, ipsilesional corticospinal tract; cCST, contralesional corticospinal tract; iCorticomotor, weighted average of the iCRST and iCST; cCorticomotor, weighted average of the cCRST and cCST.

Significant between‐group differences in motor tract strength were found for all four motor tracts assessed. When compared to controls, participants with stroke demonstrated decreased motor tract strength of the iCRST and iCST and increased strength of the cCRST and cCST (Table 2 and Figure 2).

**TABLE 2:** Stroke versus control motor tract strength.

Tract	Stroke (*N* = 10), nQA	Control (*N* = 10), nQA	Stroke − control, nQA	Stroke (*N* = 10), *z*‐score
iCRST	2.20 ± 0.45	3.41 ± 0.14	−1.21 [−1.52, −0.90]	−8.59 [−10.87, −6.32]
cCRST	3.43 ± 0.37	3.09 ± 0.22	0.34 [0.05, 0.62]	1.52 [0.34, 2.69]
iCST	2.73 ± 0.75	5.11 ± 0.24	−2.37 [−2.90, −1.85]	−9.78 [−11.98, −7.58]
cCST	5.55 ± 0.75	4.86 ± 0.24	0.69 [0.16, 1.21]	2.87 [0.63, 5.12]
iCorticomotor	2.39 ± 0.49	4.02 ± 0.14	−1.63 [−1.97, −1.29]	−11.25 [−13.65, −8.86]
cCorticomotor	4.20 ± 0.48	3.73 ± 0.17	0.46 [0.13, 0.80]	2.75 [0.73, 4.77]

*Note*: Normalized quantitative anisotropy (nQA) values for stroke and control groups are mean ± standard deviation QA values as a multiple of the mean QA in the ventricular cerebrospinal fluid. Stroke − control differences are model estimates [95% confidence interval]. Stroke *z*‐scores were calculated based on the mean and standard deviation in the control group and represent the number of standard deviations above or below the control mean value for that tract.

Abbreviations: cCorticomotor, weighted average of cCRST and cCST; cCRST, contralesional corticoreticulospinal tract; cCST, contralesional corticospinal tract; CRST, corticoreticulospinal tract; CST, corticospinal tract; iCorticomotor, weighted average of iCRST and iCST; iCRST, ipsilesional corticoreticulospinal tract; iCST, ipsilesional corticospinal tract.

**FIGURE 3:**
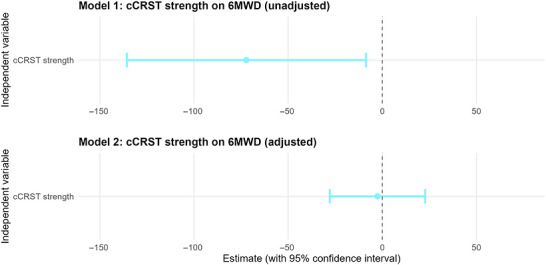
Linear regression of cCRST on 6MWD. Abbreviations: cCRST, contralesional corticoreticulospinal tract; 6MWD, six‐min walk distance

When testing whether cCRST upregulation magnitude relates to ipsilesional motor tract strength, linear regression showed a significant negative association between ipsilesional motor tract strength and cCRST strength (−0.12 SDs [−0.23, −0.02]). When testing whether the relationship between cCRST upregulation and walking function is confounded by ipsilesional motor tract strength, the unadjusted linear regression showed a significant negative association between the 6MWD and cCRST strength. This negative association was no longer present when adjusting for ipsilesional motor tract strength (Table 3 and Figure 3).

**TABLE 3:** Linear regression of cCRST strength on 6MWD.

Model 1: Unadjusted for ipsilesional damage				
Variable	Estimate	SE	*t* value	Pr(>|*t*|)
(Intercept)	401.38	50.73	7.91	0.00
cCRST strength	−72.17	30.21	−2.39	0.03
Residual standard error: 202.4 on 18 degrees of freedom, multiple *R*‐squared: 0.2407 (adjusted *R*‐squared: 0.1985), *F*‐statistic: 5.706 on 1 and 18 degrees of freedom, *p*‐value: 0.02807				

Model 2: Adjusted for ipsilesional damage				
Variable	Estimate	SE	*t* value	Pr(>|*t*|)
(Intercept)	541.44	21.23	25.50	0.00
cCRST strength	−2.56	12.01	−0.21	0.83
iCorticomotor strength	34.26	2.95	11.61	0.00
Residual standard error: 69.72 on 17 degrees of freedom, multiple *R*‐squared: 0.9149 (adjusted *R*‐squared: 0.9049), *F*‐statistic: 91.43 on 2 and 17 degrees of freedom, *p*‐value: 7.994 × 10^−10^				

Abbreviations: 6MWD, 6‐min walk distance; cCRST, contralesional corticoreticulospinal tract; iCorticomotor strength, weighted average of the ipsilesional corticoreticulospinal tract and ipsilesional corticospinal tract.

Influence diagnostics identified one participant with an elevated Cook's distance (1.45) exceeding the specified threshold of 0.821. In the leave‐one‐out sensitivity analysis, removing this participant changed the adjusted cCRST regression coefficient from −2.56 to 18.59, providing even stronger evidence of confounding. This adjusted association was still nonsignificant (*p* < 0.0001).

## 4 Discussion

This study sought to clarify whether cCRST upregulation represents a compensatory response to stroke. Compared with age‐ and sex‐matched controls, participants with stroke had significantly lower strength of the ipsilesional motor tracts (iCorticomotor, iCRST, and iCST) and significantly higher strength of the analogous tracts in the contralesional hemisphere. The lower strength of ipsilesional tracts presumably reflects damage from the stroke, while the higher strength of contralesional tracts presumably indicates upregulation of those pathways.

When assessing whether cCRST upregulation magnitude relates to ipsilesional motor tract strength, we found that lower ipsilesional strength was significantly associated with greater cCRST strength. This finding is consistent with the possibility that increased cCRST strength may be a compensation for ipsilesional motor tract damage. The predominant ipsilateral projections from the cCRST to the paretic side of the body provide an anatomical substrate for this potential compensatory role (Boyne, DiFrancesco, et al. 2022; Boyne et al. 2025). Our findings may help explain the cCRST upregulation observed in prior studies (Jang et al. 2013; Srivastava et al. 2022; Karbasforoushan et al. 2019) and extend prior knowledge by demonstrating a scaled relationship between the extent of ipsilesional damage and cCRST upregulation.

We also found evidence that the association between increased cCRST strength and walking function is confounded by the residual strength of the ipsilesional motor pathways. Similar to Srivastava et al. (2022), our unadjusted model showed that greater cCRST strength was associated with worse walking capacity (lower 6MWD). However, we found that this negative association was no longer present when adjusting for ipsilesional motor tract strength (see Table 3 and Figure 3), which is the hallmark sign of confounding. This finding directly addresses the ongoing debate over whether cCRST upregulation is adaptive or maladaptive for walking function, suggesting that it may not be maladaptive as once thought. Our finding is also consistent with a prior study, which found greater estimated cCRST volume among ambulatory versus nonambulatory participants (Jang et al. 2013). Considering our current results, this positive association between cCRST upregulation and walking independence in this prior study might have been observed because only individuals with complete damage of the iCST were included. This would partially control for ipsilesional motor tract strength (albeit not in the same way as the current study), allowing more accurate assessment of the effect of cCRST upregulation on walking independence. Taken together with the current results, these findings suggest that cCRST upregulation is not broadly harmful to walking function. Instead, cCRST upregulation may reflect an attempt by the brain to maintain function when the ipsilesional motor system is compromised.

These findings also align with the bimodal balance recovery model proposed by Di Pino et al. (2014). According to this model, the functional role of the contralesional hemisphere, such as cCRST compensation, depends on ipsilesional motor tract strength (Di Pino et al. 2014). Our data support this framework by showing that increased strength of the cCRST scales with the extent of ipsilesional damage, and that increased cCRST strength was not independently associated with poorer walking function after accounting for the extent of damage to the ipsilesional motor tracts. If this model holds with further testing, it suggests the need for individualized neurorehabilitation strategies tailored to each patient and may support targeting contralesional motor pathways in patients with lower ipsilesional motor tract strength.

More specifically, current NIBS approaches often aim to inhibit contralesional motor pathways (Veldema and Gharabaghi 2022; Di Pino et al. 2014; Plow et al. 2016). However, accumulating evidence in the upper extremity (Di Pino et al. 2014; Plow et al. 2016; Sankarasubramanian et al. 2017) suggests that facilitating contralesional motor pathways may be beneficial in individuals with great ipsilesional damage. Our findings support this possibility by suggesting that increased cCRST strength may not be maladaptive. Future NIBS studies are needed, specifically focusing on LE and walking function, to further assess the effects of facilitation of the cCRST for individuals with greater ipsilesional damage.

### 4.1 Limitations

One limitation of this study is the limited generalizability due to the small sample size. Relatedly, sensitivity analysis, including additional covariates (e.g., lesion volume), was not feasible. Furthermore, the low mean ipsilesional tract strength and walking capacity in the study sample may limit the generalizability of the findings to individuals with milder stroke. Finally, the cross‐sectional design precludes causal inference. Thus, larger longitudinal studies are needed to confirm our findings and further assess the impact of cCRST compensation on walking outcomes in patients after stroke.

## 5 Conclusion

This study demonstrates that greater ipsilesional motor tract damage is associated with increased cCRST strength. We also found that the negative association between cCRST strength and worse walking capacity was explained by the extent of residual ipsilesional motor tract strength. Therefore, our findings suggest that cCRST upregulation is not inherently harmful to walking function after stroke and may be an adaptive neuroplastic response to maximize function when ipsilesional motor pathways are more damaged. Larger studies are needed to better understand the impact of cCRST upregulation on walking function in chronic stroke.

## Author Contributions

**Jolene Foster**: Methodology, software, formal analysis, visualization, writing – original draft, writing – review and editing. **Oluwole O. Awosika**: Methodology, writing – review and editing. **Pierce Boyne**: Conceptualization, data curation, funding acquisition, investigation, methodology, project administration, resources, software, validation, visualization, writing – review and editing, supervision. All authors contributed to the article and approved the submitted version.

## Funding

This work was supported by the National Institutes of Health (grant numbers KL2TR001426, UL1TR001425, R01HD093694) and the American Heart Association (grant number 17MCPRP33670446).

## Ethics Statement

Written informed consent was obtained from all participants before the experiment, and the study protocol was approved by the University of Cincinnati IRB # 2016‐1916.

## Conflicts of Interest

The authors declare no conflicts of interest.

## Data Availability

The data that support the findings of this study are available from the corresponding author upon reasonable request.
